# Herbal Supplement in a Buffer for Dry Eye Syndrome Treatment

**DOI:** 10.3390/ijms18081697

**Published:** 2017-08-03

**Authors:** Hung-Chang Chen, Zhi-Yu Chen, Tsung-Jen Wang, Victor J. Drew, Ching-Li Tseng, Hsu-Wei Fang, Feng-Huei Lin

**Affiliations:** 1 Department of Chemical Engineering and Biotechnology, National Taipei University of Technology, Taipei 10608, Taiwan; Danielchen995@gmail.com; 2 Graduate Institute of Biomedical Materials and Tissue Engineering, College of Biomedical Engineering, Taipei Medical University, Taipei 11031, Taiwan; d79010340217@gmail.com; 3 Department of Ophthalmology, Taipei Medical University Hospital, Taipei 11031, Taiwan; tjw@tmu.edu.tw; 4 Department of Ophthalmology, School of Medicine, College of Medicine, Taipei Medical University, Taipei 11031, Taiwan; 5 International Ph.D. Program in Biomedical Engineering, College of Biomedical Engineering, Taipei Medical University, Taipei 11031, Taiwan; globalvictor87@gmail.com; 6 Institute of Biomedical Engineering and Nanomedicine, National Health Research Institutes, Miaoli 35053, Taiwan; double@ntu.edu.tw; 7 Institute of Biomedical Engineering, National Taiwan University, Taipei 10051, Taiwan

**Keywords:** dry eye syndrome, herbal extraction, kaempferol, ferulic acid, anti-inflammation

## Abstract

Dry eye syndrome (DES) is one of the most common types of ocular diseases. There is a major need to treat DES in a simple yet efficient way. Artificial tears (AT) are the most commonly used agents for treating DES, but are not very effective. Herbal extractions of ferulic acid (FA), an anti-oxidant agent, and kaempferol (KM), an anti-inflammatory reagent, were added to buffer solution (BS) to replace ATs for DES treatment. The cytotoxicity and anti-inflammatory effects were examined in vitro by co-culture with human corneal epithelial cells (HCECs) to obtain the optimal concentration of KM and FA for treating HCECs. Physical properties of BS, such as pH value, osmolality, and refractive index were also examined. Then, rabbits with DES were used for therapeutic evaluation. Tear production, corneal damage, and ocular irritation in rabbits’ eyes were examined. The non-toxic concentrations of KM and FA for HCEC cultivation over 3 days were 1 µM and 100 µM, respectively. Live/dead stain results also show non-toxicity of KM and FA for treating HCECs. Lipopolysaccharide-stimulated HCECs in inflammatory conditions treated with 100 µM FA and 1 µM KM (FA100/KM1) showed lower *IL-1B*, *IL-6*, *IL-8*, and *TNFα* expression when examined by real-time PCR. The BS with FA100/KM1 had neutral pH, and a similar osmolality and refractive index to human tears. Topical delivery of BS + FA100/KM1 showed no irritation to rabbit eyes. The corneal thickness in the BS + FA100/KM1 treated group was comparable to normal eyes. Results of DES rabbits treated with BS + FA100/KM1 showed less corneal epithelial damage and higher tear volume than the normal group. In conclusion, we showed that the combination of FA (100 µM) and KM (1 µM) towards treating inflamed HCECs had an anti-inflammatory effect, and it is effective in treating DES rabbits when BS is added in combination with these two herbal supplements and used as a topical eye drop.

## 1. Introduction

Dry eye syndrome (DES) is a multifactorial disease of the ocular surface resulting from poor tear production or retention and is associated with discomfort, burning sensations, blurry vision, visual disturbance, tear film instability, and potential damage to the ocular surface [1]. It is estimated that 100 million people worldwide may be affected by DES [2]. It has also been reported that global dry eye prevalence rates are between 5% and 34% [2], thus representing one of the most common eye diseases. DES also affects about 15% adults in the USA [3]. The DES population is rising in Taiwan due to the increased life expectancy. A study in Taiwan considered symptoms and clinical signs in 2038 participants ages 65 and over and from their symptoms alone, 33.7% had been diagnosed with dry eye [4]. Therefore, effective treatment for DES has become an issue requiring increased attention. DES is associated with inflammation of the ocular surface and hyperosmolarity of tears has been identified as being a significant contributing factor to ocular surface inflammation [1,5]. Patients suffering from DES have been observed as exhibiting an overexpression of proinflammatory cytokines on the ocular surface [6,7]. Flavonoids have demonstrated potential in the prevention and treatment of ocular disorders [8,9]. In our previous study, we found that when added to artificial tear solution, epigallocatechin gallate (EGCG), a major flavonoid of green tea, effectively inhibits the inflammation associated with DES in rabbits [10]. Kaempferol (3,5,7-trihydroxy-2-(4-hydroxyphenyl)-4*H*-1-benzopyran-4-one) (KM), another flavonoid found in many edible plants and botanical products commonly used in traditional Chinese medicine (e.g., *Ginkgo biloba* and propolis etc.) [11], has also been reported to have anti-inflammatory properties [12]. KM exhibited a protective effect on lipopolysaccharide (LPS)-induced acute lung injury in mice, and inhibits the expression of tumor necrosis factor (*TNF)-α*, Interleukin (IL)-6, and IL-8 via the modulation of the nuclear factor-κB (NF-κB) and mitogen-activated protein kinase (MAPK) pathways, making it a potential agent to be used in treating inflammatory diseases such as DES [13,14].

Eye drops are popular because they are patient-friendly and cost-effective. However, one vial of eye drops usually contains ~10 mL solution, which includes a preservative agent allowing for repeated use. Certain preservative agents have demonstrated toxicity to eyes; for example, benzalkonium chloride (BAC), a frequently used preservative utilized in ophthalmic agents, is a quaternary ammonium compound shown to hasten the drying of tear film [15], worsen pre-existing dry eye conditions, and negatively affect both the cornea and the conjunctiva [16]. An alternative method replacing preservative addition is needed. Traditional Chinese herbal therapies have drawn increased attention in recent years for modern medical/pharmaceutical applications. Among them, ferulic acid (FA), an active compound found in the angelica root (Dung-Gui), is proven to possess excellent antioxidant properties and low cytotoxicity [17]. Additionally, it has also been proven to possess an antimicrobial effect against Gram-negative and Gram-positive bacteria, as well as yeasts [18,19]. FA has shown a strong inhibitory effect against the *Escherichia coli* [19]. Therefore, we suspect it could be used as an antimicrobial agent in eye drops as a substitute for BAC.

In this study, a buffer solution (BS) is used as the basal medium for eye drops preparation. After BS preparation, we added herbal extractions of KM and FA, using the combined solutions as eye drops for DES treatment. The cell viability and anti-inflammatory effect of KM or FA for human corneal epithelial cells (HCECs) were examined; the antibacterial function of FA was also checked. Finally, the therapeutic effect of this new eye drop formula was evaluated via Schirmer’s test, fluorescence staining, corneal thickness, and histological examination of the cornea of DES rabbits treated with variant BS with or without KM/FA addition.

## 2. Results

### 2.1. Optimal Kaempferol and Ferulic Acid Concentration for Cultivating Human Corneal Epithelial Cells

**Figure 1:**
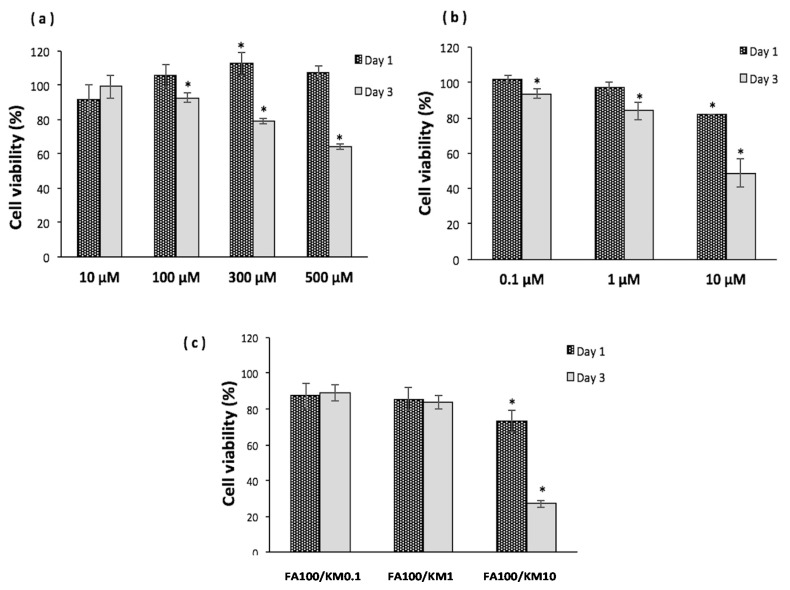
Cell viability of human corneal epithelial cells (HCECs) after incubation with varying concentrations of (**a**) ferulic acid (FA), (**b**) kaempferol (KM) and (**c**) a FA/KM mixture for one or three days. Data were analyzed using the paired t-test and are expressed as the mean ± standard deviation (SD); *n* = 6, (* *p* < 0.05 compared with the control group). FA100: 100 µM FA; KM1: 1 µM KM; KM0.1: 0.1 µM KM.

To evaluate the possible cytotoxic effect of KM and FA, the viability of HCECs was examined in the presence of KM or FA using a water-soluble tetrazolium-8 (WST-8) assay. No toxicity was observed in cells after a one-day culture with FA, even at very high concentrations (500 µM, Figure 1a). After three days of cultivation, 92.7% of cells were still alive at the FA concentration of 100 µM (Figure 1a), so we selected 100 µM of FA as a concentration for further testing. In the KM-treated group, the safety concentration is much lower than in the FA-treated group, and it is within the range of 0.1–10 µM. No significant toxicity was observed in HCECs when cultured in KM at 1 µM for 3 days, and at these dosages, the percentage of viable cells was greater than 80% (Figure 1b). However, it became toxic to HCECs when KM concentration reached 10 µM after three days of cultivation (cell viability down to 48%, * *p* < 0.05). The cell viability of HCECs treated with 100-µM FA mixed with different concentrations of KM is shown in Figure 1c. The herbal combination was non-toxic to cells at 1 µM KM with 100 µM FA (abbreviated as FA100/KM1), but many cells died with the addition of 10 µM KM; cell viability decreased to 27% (* *p* < 0.05).

**Figure 2:**
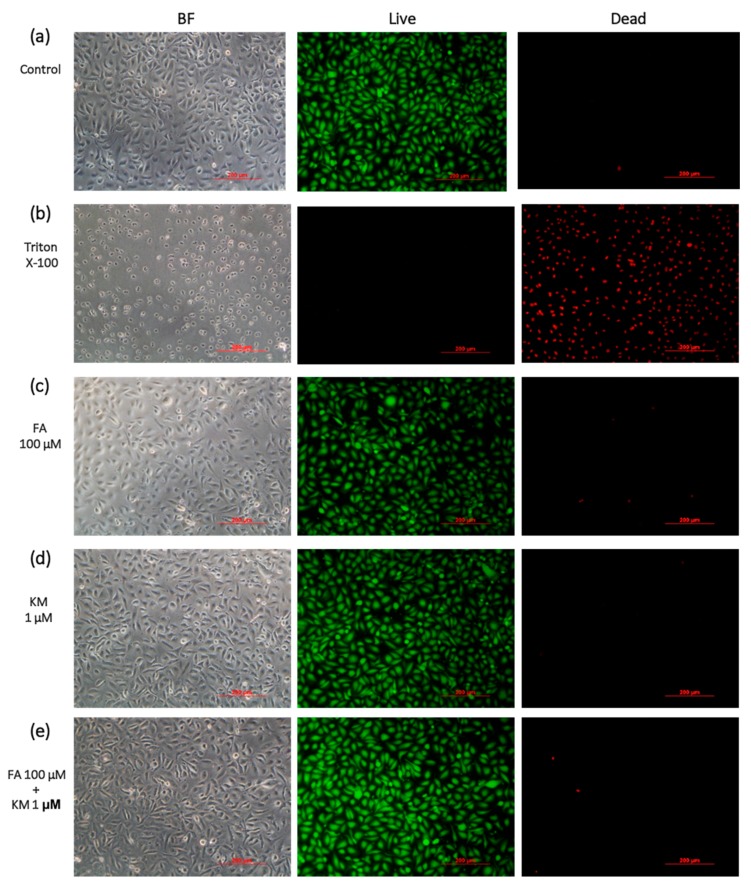
Live/dead staining images acquired from HCECs treated with (**a**) culture medium as the normal group, (**b**) Triton X-100 as a negative control, (**c**) FA at 100 μM, (**d**) KM at 1 μM, and their combination (**e**) FA/KM at 100/1 μM after one-day cultivation. BF: bright field; green: live cells; red: dead cells (Scale bare: 200 µm).

HCEC condition was also confirmed by live/dead staining on treatment with FA100/KM1 (Figure 2). Most cells revealed green fluorescence representing live cells with very low contents of red spots (dead cells), indicating that the FA100/KM1 solution was not harmful to HCECs at this concentration.

### 2.2. The Anti-Inflammatory Effects of KM and FA Work on Inflamed HCECs

**Figure 3:**
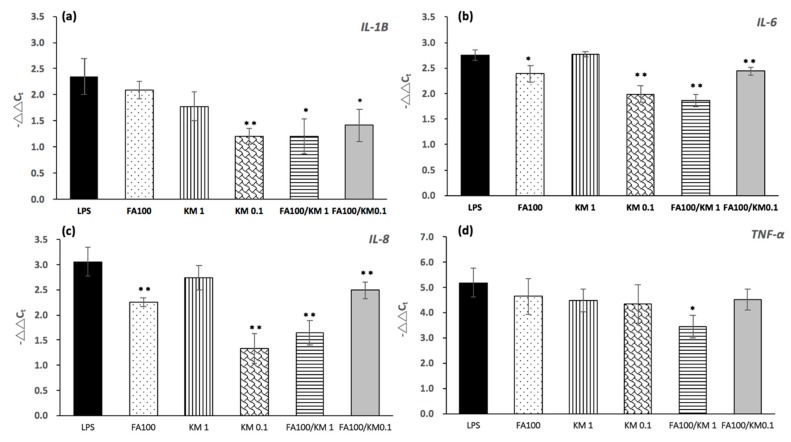
Gene expression of (**a**) *IL-1B*, (**b**) *IL-6*, (**c**) *IL-8*, and (**d**) *TNFα* in HCECs showing variations in LPS-induced inflammation (6 h) following various treatments. The control group consisted of cells left untreated with LPS. Results are displayed as the fold increase compared to the expression in normal HCECs. Statistical analysis was taken for each group in comparison with the LPS group (*n* = 6, * *p* < 0.05, ** *p* < 0.01). LPS: lipopolysaccharide; FA100: 100 µM FA; KM1/KM0.1: 1/0.1 µM of KM; FA100/KM1: 100 µM FA + 1 µM KM; FA100/KM0.1: 100 µM FA + 0.1 µM KM.

We examined the anti-inflammatory effect of KM, FA or their combination on LPS-stimulated HCECs. Figure 3 shows the expression levels of *IL-1B*, *IL-6*, *IL-8*, and *TNFα* in inflamed HCECs incubated with LPS (500 ng/mL), FA100, KM1, 0.1 μM KM (KM0.1), FA100/KM1, and FA100/KM0.1. Figure 3 shows a comparison of these conditions to the normal baseline levels of HCECs. LPS-treated HCECs showed high expression of *IL-1B*, *IL-6*, *IL-8*, and *TNFα*. The levels of *IL-1B*, *IL-6*, and *IL-8* were significantly downregulated in cells treated with KM0.1, FA100/KM1 and FA100/KM0.1 (* *p* < 0.05). The expression of *TNFα* was only significantly inhibited by cells treated with FA100/KM1 (* *p* < 0.05). According to these results, the optimal concentrations for inflammation inhibition of HCECs using an FA/KM combination were 100 µM and 1 µM, respectively.

### 2.3. Characterization of Buffer Solution Containing FA and KM as Eye Drops

In normal human tears, the pH value varies between 6.5–7.6 [20], and the osmolarity is about 260~340 mOsm/kg [21]. The pH of BS, BS + FA100 or BS + KM1 was from 7.15 to 7.25, and the range of osmolarity was between 301–307 mOsm/kg (Table 1). The BS + FA100/KM1 had a pH value at 7.25 ± 0.15, and its osmolarity was 302.0 ± 1.0 mOsm/kg. The refractive index was approximately 1.3345, similar to that of normal human tears (1.33698 ± 0.0011) [22]. These data show that BS with FA/KM addition mimics normal human tears.

### 2.4. Irritation Tests in Rabbit Eyes

**Figure 4:**
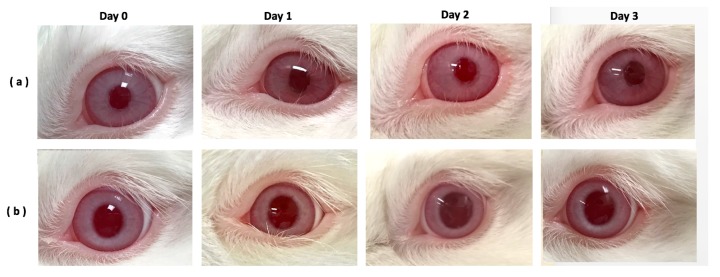
The appearance of rabbit eyes treated with 50 µL of (**a**) BS (*n* = 3) and (**b**) BS with FA100/KM1 (*n* = 8) addition using topical delivery as eye drops acquired on day 1 to day 3.

The rabbit eye irritation test was developed to predict ocular irritancy in humans. We performed an irritation test on rabbits after a single instillation of 50 µL of BS with FA100/KM1. Eyes treated by BS were used as a control. Each animal was observed at one, two, and three days after instillation. The grade and record of the ocular lessions were based on the International Organization for Stndardization (ISO) 10993-10:2010(E) [23]. There was no severe blinking of rabbit eyes observed under a slit lamp examination. Intensive tear secretion revealed discomfort in rabbits eyes was not found; also no opacity was observed in the cornea. No redness, chemosis, or discharge were found in the conjunctiva. Edema and haemorrhage were also not observed (Figure 4). No differences were observed in the ocular tissue of rabbits treated with BS or BS plus FA100/KM1. Studies have shown that rabbit eyes are more sensitive than human eyes, and they take longer for epithelial repair [24,25]. According to this, it is speculated that BS with FA100/KM1 addition is tolerated and would result in no irritation in human eyes.

### 2.5. Therapeutic Efficacy of Buffer with FA/KM Addition in DES Rabbits

**Figure 5:**
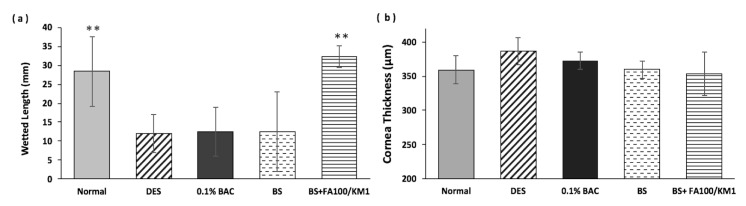
Results of (**a**) Schirmer’s tests for tear production, (**b**) corneal thickness variation in all treated groups. All groups were compared to the dry eye syndrome (DES) group for statistical analysis (*n* = 4, ** *p* < 0.01).

**Figure 6:**
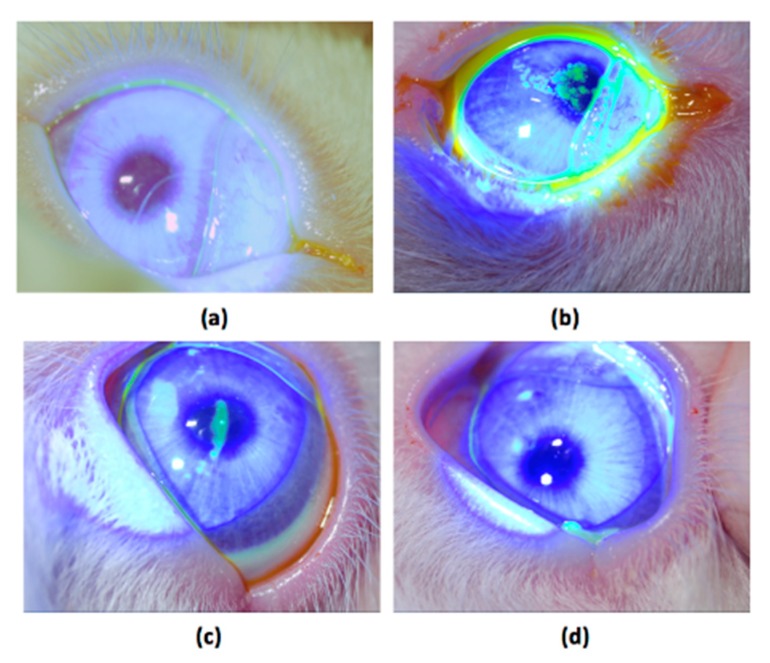
Slit lamp photographs of rabbit eyes after fluorescein (FL) staining. (**a**) Normal, (**b**) 0.1% benzalkonium chloride (BAC), (**c**) BS, and (**d**) BS plus FA100/KM1 treated groups. Damaged epithelial cells in the eyes were observed as green patches.

**Figure 7:**
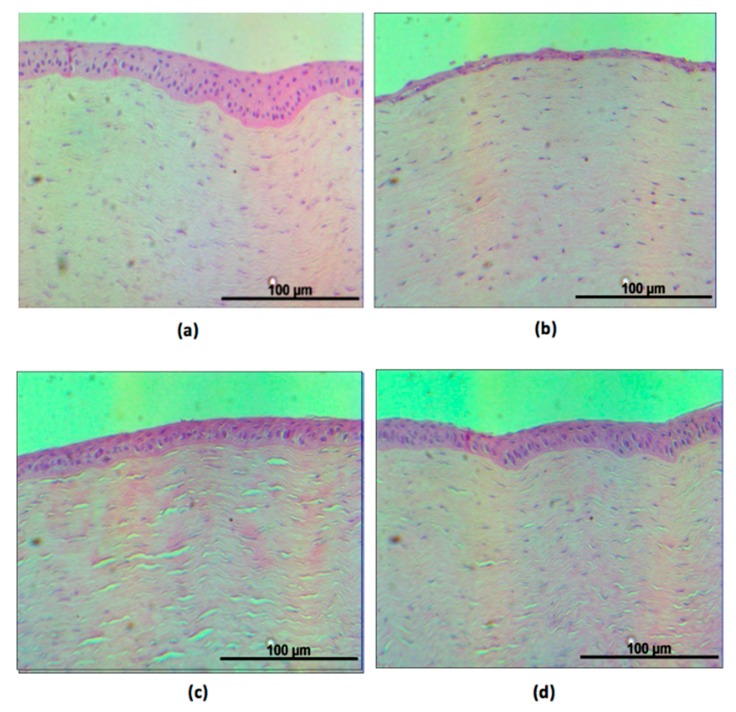
Representative pictures of hematoxylin and eosin (HE)-stained corneal sections from the eyes of rabbits in variant groups. (**a**) Normal cornea, (**b**) 0.1% BAC, (**c**) BS, and (**d**) BS + FA100/KM1.

The timeline from DES induction to treatment was described as follows: at the beginning, rabbits were treated with 0.1% BAC three times every day for four weeks in order to induce DES in the rabbits’ eyes. As rabbits developed clinical signs that mimicked DES, the rabbits’ eyes were treated with variant eye drops made with BS or BS plus FA100/KM1 thrice daily lasting for three weeks. Figure 5, Figure 6 and Figure 7 show the DES group of rabbits treated with 0.1% BAC for four weeks; the 0.1% BAC group shown in these figures received another three weeks of treatment with 0.1% BAC (in total seven weeks of treatment with 0.1% BAC).

#### 2.5.1. Tear Production Increased on Treatment with the Herbal Combination

Tear production was examined after the three weeks of treatment with the variant BS as shown in Figure 5a. Compared with the normal group (wetted length: 28 ± 9 mm), tear secretion on treatment with 0.1% BAC for four weeks resembled DES, confirming successful model establishment; the wetted length was reduced rapidly, decreasing to 12 ± 5 mm. After another three weeks of treatment of 0.1% BAC there were similar results, where the tear volume was lower than in the normal group. After a three-week treatment period, the tear secretion in the BS group was approximately 13 ± 11 mm, showing no effectiveness towards the treatment of DES. A statistically significant increase was observed in the group treated with BS + FA100/KM1, yielding a tear volume of 32 ± 3 mm (Figure 5a, (** *p* < 0.01). The corneal thickness is shown in Figure 5b. Compared to the control groups (360 ± 21 µm), results of DES or 0.1% BAC revealed an increase in corneal thickness, reaching above 375 µm. After three weeks of treatment with BS or BS plus FA/KM, corneal thickness was almost the same as the control group (~355 µm).

#### 2.5.2. Recovering the Damaged Epithelium in the Cornea

Following the three-week treatment, the ocular surfaces of each experimental group excluding the eyes of normal animals were stained using fluorescein (FL) and observed through slit lamp microscopy (Figure 6a). In contrast, fluorescence green staining was used to examine the center of the cornea with 0.1% BAC in another three-week treatment (Figure 6b). A light green stain in the center cornea can still be observed in the BS-treated eye (Figure 6c), but no green staining was found in the BS + FA100/KM1-treated eye (Figure 6d).

Histological examination under a light microscope showed that the normal rabbit corneas had multilayered epithelial cells (three to five layers) and a dense stromal layer (Figure 7a). Rabbits in the 0.1% BAC group had a thinner corneal epithelium, with only one to two layers (Figure 7b). Although the epithelial layers in the BS group seemed to exhibit normal recovery, a very loose collagen structure in the stroma was observed (Figure 7c). The rabbit corneas in the BS + FA100/KM1 group revealed multilayered corneal epithelium and a common stroma structure as found in normal cornea (Figure 7d). No obvious signs of inflammation or vessel formation in the cornea were observed from histological examination of the rabbit eyes.

## 3. Discussion

Inflammation and tear hyperosmolarity have been described as symptoms of DES [26], and studies about anti-inflammatory treatment of DES have been thoroughly investigated [5,27,28]. In this study, we confirm that the herbal extraction combination consisting of kaempferol (KM, anti-inflammatory agent) and ferulic acid (FA, predicted antibacterial) has a therapeutic effect in the relief of certain DES symptoms in rabbits.

To determine whether these herbal extractions affect the viability of cells, a WST-8 assay was performed on HCECs treated with KM or FA at variant concentrations for one and three days. Crespo et al. revealed that human umbilical vein endothelial cells (HUVECs) cultured with 10 µM KM for one day did not significantly decrease cell viability [29]. We observed a similar tendency in that HCECs cultured in medium with a 10-µM KM supplement still had cell viability above 80% at day one. A study conducted by Sharma et al. revealed that cell viability of glioblastoma cell lines was around 60% after 72-h culture with KM at a concentration of 50 µM in medium [30]. In this study, HCECs were more sensitive than glioblastoma cells; 10 µM of KM caused HCEC viability to drop to 50% after three days of cultivation. According to literature review, there is no related study on the application of KM to HCECs, making this the first attempt in using KM for cornea epithelial cells or related ocular disease treatment. The safest KM concentration for treating HCECs was 1 µM, proven by WST-8 and live/dead staining assays (Figure 1 and Figure 2). Basically, stocked KM solution was dissolved in dimethyl sulfoxide (DMSO). We also confirmed the solvent effect for influencing HCECs. When DMSO concentration <0.1% (*v*/*v*), it is nontoxic to HCECs; cell viability was 83.0 ± 2.5% (data not shown). When KM concentrations were increased to 10 µM, the final DMSO concentration in medium was <0.05%. Therefore, the toxicity effect resulted from KM, not DMSO (Figure 1 and Figure 2).

As mentioned, DES can be characterized by increased levels of inflammation on the ocular surface [31]. Toll-like receptors (TLRs) are the earliest detectors of the immune system replying to inflammatory activities in many tissues including the eyes [31]. One of them is TLR4, a toll-like receptor subtype activated by lipopolysaccharide (LPS), which is a component of gram-negative bacteria. Therefore, LPS can activate TLR4 leading to the production of inflammatory cytokines, such as *IL-1β* and *TNF-α* in cells [32,33]. Cao et al. demonstrated that LPS showed eye irritation and inflammatory potential in vitro; the HCE tissue culture with LPS exposure can be used as an in vitro model to study environmental exposure-induced eye irritation and inflammation [34]. LPS-induced inflammatory HCECs treated with EGCG (10 µg/mL) and hyaluronic acid (HA) (0.1% (*w*/*v*) HA) combination solution significantly inhibited the expression of *IL-1β*, *IL-6*, *IL-8*, and *TNFα* in inflamed HCECs [10]. In this study, another flavonoid, kaempferol, is also known to possess anti-inflammatory properties and thus has therapeutic potential for the treatment of inflammatory diseases such DES. To date, the effect of KM toward inhibiting cornea epithelium cell inflammation is unknown. We identified the anti-inflammatory effect of not only KM, but also the synthetic effect of KM combined with FA on inflamed HCECs induced by LPS (Figure 3). Higher concentrations (FA 100 µM or KM 1 µM) have no effect on inflammatory gene inhibition, although they demonstrate non-toxicity to HCECs (Figure 1). When KM is present at low concentrations (0.1 µM), it significantly suppressed the inflammatory gene expressions of *IL-1B*, *IL-6*, and *IL-8*, but not *TNFα*. When KM + FA were present at concentrations of 1 and 100 µM individually, they significantly inhibited *IL-1B*, *IL-6*, *IL-8*, and also *TNFα* expression in inflamed HCECs (Figure 3). The anti-inflammatory effect of KM was studied showing that using 12.5 and 25 μg/mL KM cultured with cardiac fibroblasts can significantly suppressed the release of TNF-α, IL-1β, IL-6, and IL-18 and inhibited activation of NF-κB and serine/threonine protein kinase (Akt) in LPS plus ATP-induced cardiac fibroblasts [35]. Certain flavonoids, such as kaempferol, express anti-inflammatory activity at least in part by modulation of pro-inflammatory gene expression such as cyclooxygenase-2 (COX-2), inducible nitric oxide (NO) synthase, and several pivotal cytokines [36]. Flavones, for example apigenin, luteolin, kaempferol, and quercetin (without the C-3-hydroxyl group) more strongly down-regulate pro-inflammatory gene expression. Additionally, kaempferol inhibited NO production by inducible nitric oxide synthase (iNOS) down-regulation [37]. It also strongly impeded COX-2 induction by inhibiting nuclear transcription factor-B (NF-κB) activation via inhibitor-B (IB) kinase inhibition [37]. Kaempferol inhibited iNOS proteins, mRNA expression and NO production in a dose-dependent manner. It also inhibited the activation of nuclear factor-κB, which is a significant transcription factor for iNOS. Kaempferol also inhibited the activation of the signal transducer and activator of transcription-1 (STAT-1), another important transcription factor for iNOS [38]. When KM inhibits inflammation on the ocular surface, it promotes corneal epithelium or goblet cell repair, then it causes an increase of mucin and tear production, reducing tear film instability, and ultimately relieving signs of DES in rabbits. KM is also a kind of antioxidant with phenolic compounds that helps by exhibiting tyrosinase inhibitory activity, iNOS activity, or alkaline phosphatase-stimulating activity in cells [13,39]. These activities often begin with interaction between KM and various proteins, especially enzymes. Through these interactions, KM can induce downstream signal transduction [40]. Based on its phenolic nucleus and unsaturated side chain, FA can readily form a resonance-stabilized phenoxyl radical structure [41]. When KM mixes with FA, FA can stabilize the KM structure, making it difficult for KM to oxidize. Thereafter, KM can interact with proteins, inducing longer durations of cascade signals for cell activities. This a possible reason why KM was more effective when mixed with FA as a combination solution than on its own.

The pH and osmolality of BS in combination with KM or FA were in a suitable range (Table 1). No ocular redness, itching or draining were observed during the treatment period (Figure 4), confirming its suitability as an eye drop for extraocular treatment. Theoretically, the addition of FA in BS in this study was based on its antibacterial effect. Its concentration (100 µM) was fixed according to the cytotoxicity test, but the 100 µM FA addition at for bacterial inhibition test may not be high enough, producing only a slight inhibition in *Escherichia coli* (ATCC25922) and having no effect on *Staphylococcus aureus* (ATCC 6538) and *Pseudomonas aeruginosa* (ATCC 11633) (data not shown). Considering the safety to HCECs, the storage duration of the BS with KM/FA and the lack of additional preservatives, providing a small volume package of BS + KM/FA for daily use is another way to solve this problem.

In this study, a buffer solution designed to mimic BSS PLUS^®^ was prepared as the base solution for FA and KM supplements for DES treatment. BSS PLUS^®^ is a type of sterile intraocular irrigating solution used for intraocular surgical procedures [42], but side effects including incidents of corneal edema, corneal decompensation and postoperative inflammatory reactions have all been associated with the use of BSS PLUS^®^ [42]. BSS PLUS^®^ is not commonly used as a topical vehicle for eye drop formulations. However, an in vivo study in rabbits has shown that BSS PLUS^®^ solution is more suitable than normal saline or balanced salt solution for intravitreal irrigation because BSS PLUS solution contains appropriate bicarbonate, pH, and ionic composition [42]. Therefore, we believe it is also suitable as the base solution to be applied in eye drop formulations. In the results of the anti-inflammation examination (Figure 3), the synthetic effect of FA100/KM1 was observed, demonstrating more effective inhibition of inflammatory gene expression (*IL-1β*, *IL-6*, *IL-8* and *TNFα*) compared with KM or FA alone. The combined formulation should be more effective in treating inflammation in DES. Due to these results, and considering the “3-R” concept for animal study, alternative methods used replacement, reduction, and refinement for animal tests. Therefore, only the combined formulation (FA100/KM1) was tested for in vivo evaluation in this study. Here, all formulations made by BSS PLUS^®^-based solution (BS) with KM or FA addition were delivered to rabbits’ eyes via eye drops, and no irritation (Figure 4) or corneal edema (Figure 4 and Figure 5b) were observed. In addition, the tear volume in the BS + FA100/KM1 group returned to the normal range (Figure 5a), and differed significantly from the 0.1% BAC- and BS-treated groups. This may result from the restoration of epithelium cells in DES rabbits. The central corneal thickness (CCT) in rabbits was around 356 ± 14 µm using an optical low coherence reflectometer (OLCR) mounted on a regular slit lamp [43]. A study using spectral-domain anterior segment optical coherence tomography (AS-OCT) to measure CCT was performed, and its results revealed that rabbit CCT was about 386 µm ± 20 µm [44]. In Figure 5b, the normal CCT in this study is in a range comparable with the previous study mentioned above, and no statistical difference between tested groups were found.

Topical delivery of BAC to induce dry eye conditions in mice, rats, rabbits, cats, and dogs were reported in previous research [45,46,47,48]. A BAC-induced rabbit DES model was chosen in this study due to its established ease to perform. BAC stimulates the overexpression of inflammatory cytokines such as intercellular adhesion molecule (ICAM)-1, IL-6, IL-8, and IL-10 in epithelial cells [49,50,51], which may promote apoptosis of both epithelial and goblet cells [50]. This loss of epithelial and goblet cells may reduce mucin expression. In turn, reduced mucin production accelerates the disruption of the tear film, thus aggravating the damaged areas of the ocular surface stimulating an inflammatory feedback cascade in the epithelial cells of the ocular surface [50]. BAC can also disrupt the cornea epithelial barrier resulting in tear film instability; its accumulation also induces a reduction of mucins and an alteration of the lipid layer, leading to impairments of the tear film with tear instability and excessive evaporation [26,52]. Reports indicate that lower concentrations of BAC led to increased damage to the conjunctiva and cornea over several weeks, ultimately producing an outcome of dry eye [52,53]. Although the BAC-induced DES rabbit model does not represent the all of the signs of DES, the phenomena caused by BAC treatment including tear film instability, tear volume reduction, and damage to cornea epithelial cells (Figure 5, Figure 6 and Figure 7) were revealed and they are among the common features used in the characterization of DES. Corneal changes in the rabbits’ eyes had a tendency to recover within two weeks after successful establishment of the dry eye model [50]. In our previous study, after four-week induction with 0.1% BAC treatment auto-recovery was not possible after halting BAC [10], so the dry eye sign relief resulted from the use of BS + FA100/KM1 and not from the withdrawal of BAC.

Here, fluorescein staining revealed heavy damage to the corneal epithelium after BAC treatment (Figure 6b). On the other hand, DES eyes treated with BS + FA100/KM1 did not demonstrate dye uptake (Figure 6d), suggesting that the damaged corneal epithelium had recovered. DES induced by BAC is a leading cause in pathological changes to the cornea, including squamous metaplasia and apoptosis, which are highly associated with inflammation [10,28,50,54]. Many studies indicate that BAC plays a significant role in the overexpression of IL1-β, IL-6, IL-8, and TNFα in epithelial cells as well as corneal protein extraction [10,49,50,51,54], which can promote apoptosis of both goblet and epithelial cells [50]. In the 0.1% BAC-treated group, the epithelium cells layer detached and became thinner; only one or two layers of epithelium lining remained (Figure 7b). Similarly to our previous study [10], DES rabbits with no effective therapeutic treatment also have loose collagen structures in cornea stroma; gaps were found in the BS group (Figure 7c). Multilayer corneal epithelium and dense stroma mimicking normal cornea were revealed in the BS + FA100/KM1 group (Figure 7d), which may be due to inflammation inhibition by KM/FA in corneal epithelium cells (Figure 4), causing the recovery cascade of epithelium cells to relieve signs of DES in the rabbits.

## 4. Materials and Methods

### 4.1. Chemicals and Reagents

Kaempferol, ferulic acid, lipopolysaccharide (LPS), glutathione, dextrose, benzalkonium chloride (BAC), hydrocortisone, Triton X-100, dimethyl sulfoxide (DMSO), Cell Counting Kit-8 (CCK-8), and live/dead cell double staining kit were obtained from Sigma-Aldrich (St. Louis, MO, USA). Chemical solutions including calcium chloride, sodium chloride, sodium bicarbonate and sodium phosphate dibasic, potassium chloride, magnesium chloride hexahydrate, and dextrose were purchased from Showa Chemical Industry (Meguro-ku, Tokyo, Japan). Keratinocyte serum-free medium (KSFM), trypsin-EDTA, bovine pituitary extract (BPE), insulin, penicillin and streptomycin, and phosphate-buffered saline solutions were acquired from Gibco BRL (Gaithersburg, MD, USA). High-Capacity complementary DNA (cDNA) Reverse Transcription Kit and the TaqMan Fast Universal Master Mix (2×) were purchased from Applied Biosystems (Foster City, CA, USA). Epithelial growth factor (EGF) was acquired from PeproTech (Rocky Hill, NJ, USA). FNC Coating Mix was obtained from Athena Environmental Sciences, Inc (Baltimore, MD, USA). Schirmer’s strips (Tear Touch) were acquired from Madhu Instruments (New Delhi, India). Zoletil 50 was purchased from Virbac Animal Health (Vauvert, Nice, France) and rompun solution (2%) was obtained from Bayer Korea, Ltd (Ansan-city, Kyonggi-do, Korea). Alcaine 0.5% Ophthalmic solution was obtained from Alcon-Couvreur N.V. (Puurs, Belgium). Fluorescein paper strips were obtained from HAAG-STREIT AG (Koniz, Switzerland; Bern, Switzerland). All other chemicals and solutions were obtained from Sigma-Aldrich.

### 4.2. Viability of Human Corneal Epithelial Cells Treated with Herbal Components

The HCEC line was obtained from American Type Culture Collection (No. CRL-11135; Manassas, VA, USA). Prior to HCEC seeding, FNC Coating Mix was used to treat tissue culture plastics. Medium for HCEC cultures contained KSFM supplemented with 5 ng/mL insulin, 5 ng/mL EGF, 50 ng/mL BPE, and 500 ng/mL hydrocortisone. The CCK-8 method was used to determine cell viability of HCECs treated with different concentrations of KM or FA solutions. These cells were seeded in 96-well plates (5 × 10^3^ cells/well) and cultured overnight. Next, the HCECs were incubated with KM (0.01–50 µM) or FA (1–500 µM) for one and three days. A high concentration of KM was dissolved in DMSO as stocked solution. Stock FA solution was prepared using deionized (DI) H_2_O as a solvent. At the scheduled time-point, the culture medium was discarded, and 200 µL of tetrazolium-8 (WST-8) working solution was added to each well. After 4 h of incubation, optical density (OD) value at 450 nm was measured and the solution was assessed by a micro spectrophotometer (Infinite M200; Tecan Trading AG, Männedorf, Switzerland). The reference wavelength was set at 650 nm. The percentage of viable cells was calculated by comparison to that of the control cells treated by culture medium only. HCECs were stained with a live/dead staining kit to observe cell viability according to the instructions. Cells emitting green fluorescence revealed live status, whereas dead cells could be stained by propidium iodide (PI) and would emit a red fluorescence. Images were acquired using an inverted fluorescence microscope (Olympus, IX81, Tokyo, Japan).

### 4.3. Expression of Genes Encoding Inflammatory Cytokines in HCECs

HCECs were seeded in 24-well plates (1 × 10^5^ cells/well) with culture medium and incubated for 2 days. In order to induce inflammation, the medium was replaced with 500 ng/mL LPS-containing fresh medium. Non-LPS-treated cells were used as control group. After LPS stimulation for 6 h, the medium was replaced with fresh medium containing KM at 0.1 and 1 µM (abbreviated as KM0.1, KM1), or FA at 1 and 100 µM (FA1, FA100), or a combination of the two. After another 2 h of cultivation, the cells were collected and the total amount of RNA was extracted using TRIzol reagent in accordance with the manufacturer’s instructions. Isolated RNA was stored at −80 °C until it was to be utilized in reverse transcription-polymerase chain reaction (RT-PCR). The RNA was adjusted to a concentration of 2 µg/10 µL, and the first strand of complementary DNA (cDNA) was synthesized via the high-capacity cDNA Reverse Transcription Kit in accordance with the manufacturer’s protocol. Real time-PCR was conducted on a StepOne Real-Time PCR System (Applied Biosystems) using a TaqMan Universal PCR Master Mix (2×) and the following primers: *TNFα* (Hs00174128m1), *IL-1B* (Hs01555413m1), *IL-6* (Hs00174131m1), *IL-8* (Hs 00174103m1), and glyceraldehyde-3-phosphate dehydrogenase (*GAPDH*; Hs99999905m1). The ΔΔ*C*_t_ method was used to quantify relative gene expression.

### 4.4. Characterization of Buffer Solution Containing Herbal Components as Eye Drops

Optimal concentration of KM and FA for treating HCECs was decided based on the result of the CCK-8 test; finally, KM1 and FA100 were supplemented in the buffer solution (BS). The chemical composition of BS is based on the balance salt solution (BSS PLUS^®^) for further animal studies. The pH and osmolarity of BS and herbal combination were determined using a pH meter (pH 510; Eutech Instruments, Singapore) and a micro-osmometer (Model 3320; Advanced Instruments, Norwood, MA, USA). A refractometer (DR-A1 ATAGO, Kyoto, Japan) was used to measure the refractive index (RI) of the solution. The BSS PLUS^®^ is composed by two aliquots: solution (A) with NaCl (3.571 g), KCl (0.189), Na_2_HPO_4_ (0.207) and NaHCO_3_ (1.051) in 480 mL; and solution (B) with CaCl_2_, MgCl_2_⋅6H_2_O, detrose, and glutathione in 20 mL [42]. Solutions A and B were freshly prepared before use; they were preservative-free and aseptic after filtration through a 0.22-µm filter. Afterwards, both solutions were mixed before use and abbreviated as BS for further animal testing in this study.

### 4.5. Irritation Test of Rabbit Eyes

Male New Zealand white rabbits with a weight range between 2.5 and 3.5 kg were used. In total, 50 µL of sterilized BS with herbal extraction was dropped using a pipet into the lower conjunctival sac of the rabbits’ eyes. The same volume of BS was administered to another group of rabbits as a control. The irritation test was performed using clinical evaluation methods examining discomfort, discharge, cornea/conjunctival chemosis, or redness, as described in using a modification of the scoring system established by ISO-10993-10 [23]. Six rabbits were used in this test. Eyes with unhealthy conditions were ruled out. Four rabbits (eight eyes) were tested in the BS + FA100/KM1 group, and three eyes from two rabbits were tested in the BS group (one eye was excluded because the ocular surface was scraped by a tip during dropping). Each animal was observed and tested at 0, 1, 2 and 3 days after instillation.

### 4.6. DES Inducement in Rabbits and Therapeutic Effect Evaluation of Herbal Eye Drops

Male New Zealand white rabbits were used for these tests. All experimental methods and procedures performed during this study were approved by the Institutional Animal Care and Use Committee (IACUC) of Taipei Medical University (IACUC approval no. LAC-2015-0131, 9 April 2015). These animals were housed in standard cages at a temperature of 23 °C ± 2 °C, relative humidity of 60 ± 10%, and a light-controlled room with a 12-h light–dark cycle. For eye drop dosing, rabbits were maintained in fixing boxes without anesthesia. The protocol for DES inducement was executed as previously described [10]. Eye drops with 0.1% (*wt*/*v*) BAC (20 µL) were administered three times daily for four weeks to induce rabbits with DES. Prior to treatment, the DES condition in the rabbits of each group was examined in the following way: clinical ocular observations followed by Schirmer’s tests; fluorescein staining was conducted both before and after BAC treatment to assess DES induction. While performing these examinations, rabbits were under whole anesthesia administered by an intramuscular injection of Zoletil 50 with 2% Rompun (1:2 ratio, 1 mL/kg). After DES inducement, rabbits were randomly divided into three groups: (1) BS, (2) BS + FA100/KM1 (FA 100 µg/mL plus KM 1 µg/mL), and (3) 0.1% (*wt*/*v*) BAC (negative control). Each group was tested on two rabbits (four eyes) treated with different eye drops (20 µL) for two batch tests (total eight rabbits). Normal rabbits’ eyes were used as positive controls. The eye drops were administered three times daily for three weeks. The rabbits were then sacrificed and their corneas were carefully dissected. 

#### 4.6.1. Measurement of Aqueous Tear Production and Fluorescence Staining

Tear amounts were measured by using Schirmer’s strips [47,54]. Rabbits were anesthetized firstly; then, following topical administration of 0.5% Alcaine^®^ eyedrops (20 µL), the lower eyelid was gently pulled down allowing for Schirmer’s strips to be placed on the palpebral conjunctival vesica. Five minutes later, the length of the wetted area of the strip was recorded (in mm). Tested results were evaluated by the same person at designated time points. Fluorescein staining of the corneas was used to confirm corneal integrity [47,55]. The therapeutic effect of variant eye drops was confirmed after the three-week treatment. The fluorescein (FL) paper strips were wiped on the ocular surface and fluorescence staining on the cornea was examined and graded under a portable slit lamp (KOWA SL-17, Kowa Company Ltd., Tokyo, Japan). Corneal thickness was measured by iPac Hand-held Pachymeter (Reichert Inc., Depew, NY, USA).

#### 4.6.2. Histological Examination of the Cornea

After sacrificing the rabbits, each cornea was fixed in a 3.7% formaldehyde buffer solution for a duration of 24 h. Next, the fixed cornea specimens were placed firmly in paraffin before being sectioned. Hematoxylin and eosin (HE) staining was used to stain the sections for histological examination. Each section was observed through an optical microscope (BM-1A; SAGE vision, New Taipei City, Taiwan).

### 4.7. Statistical Analysis

All data are displayed as an average ± standard deviation (SD) from two to three independent experiments. Student’s *t*-tests were performed using SPSS 17.0 (SPSS, Inc., Chicago, IL, USA) and one-way analysis of variance (ANOVA) was used to assay statistical differences between groups. A probability value (*p*-value) of 0.05 was considered statistically significant.

## 5. Conclusions

In this study, FA at 100 µM and KM at 0.1 µM were nontoxic to HCECs and could also inhibit the expression of inflammatory genes such as *IL-1B*, *IL-6*, *IL-8*, and *TNFα* in inflamed HCECs. Characterization of BS containing FA100/KM1 showed that it mimicked human tears, with a similar pH, osmolarity, and refractive index. Topical administration of BS + FA100/KM1 can relieve some DES signs such as tear production and cornea repair with a noticeable therapeutic effect in rabbits, but the change in goblet cells and real-time anti-inflammation effect in the eyes will need to be clarified in future work. A limitation of the herbal component FA is that its presumed antibacterial function is not confirmed in this study due to its low concentration and non-effectiveness in inhibiting bacterial growth. Further clinical investigations are needed to assess the efficacy and safety of this eye drop for treating DES in humans in the future.

## Figures and Tables

**Table 1:** Characteristics of buffer solution (BS) with varying herbal combinations.

Group	pH	Osmotic Pressure (mOsm/kg)	Refractive Index (RI)
Normal human tears	6.5–7.6 [20]	260–340 [21]	1.3369 ± 0.0011 [22]
BS	7.15 ± 0.05	301.3 ± 0.6	1.3345 ± 0.0001
BS + FA100	7.20 ± 0.02	306.7 ± 1.5	1.3345 ± 0.0001
BS + KM1	7.20 ± 0.04	301.7 ± 1.2	1.3344 ± 0.0001
BS + FA100/KM1	7.25 ± 0.15	302.0 ± 1.0	1.3344 ± 0.0000

FA100: 100 µM FA; KM1: 1 µM KM; FA100/KM1: 100 µM FA + 1 µM KM mixture.

## Abbreviations

DES	Dry eye syndrome
BS	Buffer solution made by balance salt solution
KM	Kaempferol
FA	Ferulic acid
BAC	Benzalkonium chloride
HCECs	Human corneal epithelium cells
